# Antifungal susceptibility patterns of colonized *Candida* species isolates from immunocompromised pediatric patients in five university hospitals

**Published:** 2017-12

**Authors:** Parisa Badiee, Maral Choopanizadeh, Abdolkarim Ghadimi Moghadam, Ali Hossaini Nasab, Hadis Jafarian, Ahmad Shamsizadeh, Jafar Soltani

**Affiliations:** 1 Prof. Alborzi Clinical Microbiology Research Center, Shiraz University of Medical Sciences, Shiraz, Iran; 2 Department of Pediatrics, University of Medical Sciences, Yasuj, Iran; 3 Department of Pediatrics, Kerman University of Medical Sciences, Kerman, Iran; 4 Infectious and Tropical Diseases Research Center, Health Research Institute, Ahvaz Jundishapur University of Medical Sciences, Ahvaz, Iran; 5 Department of Pediatrics, Besat Tertiary Hospital, Kurdistan University of Medical Sciences, Sanandaj, Iran

**Keywords:** *Candida* species, Colonized, Amphotericin B, Voriconazole, Posaconazole, Itraconazole

## Abstract

**Background and Objectives:**

Colonization of *Candida* species is common in pediatric patients admitted to hematology-oncology wards. The aim of this study was to identify colonized *Candida* species and their susceptibility patterns in hematologic pediatric patients.

**Materials and Methods:**

Samples were collected from mouth, nose, urine and stool of the patients admitted to five university hospitals and cultured on sabouraud dextrose agar. The isolates were identified by API 20 C AUX system and their susceptibility patterns were evaluated by CLSI M27-A3 and S4.

**Results:**

From 650 patients, 320 (49.2%) were colonized with 387 *Candida* species. *Candida albicans* was the most prevalent isolated species, followed by *Candida glabrata, Candida tropicalis, Candida famata, Candida kefyr* and *Candida kuresi*. The epidemiological cut off value (ECV) for all *Candida* species to amphotericin B was ≤0.25 μg except *C. krusei* (4 μg). The resistance rate to fluconazole in this study in *C. albicans* was 4.9% with ECV 8 μg/ml, followed by *C. tropicalis* 8.8% with ECV 0.5 μg/ml. Voriconazole and posaconazole were effective antifungal agents for all *Candida* isolates. The ECV of *C. albicans, Candida parapsilosis, C. tropicalis, C. glabrata* and *C. krusei* for itraconazole were 0.5, 0.25, 0.5, 1 and 2 μg, respectively. The resistant and intermediate rates of *Candida* species to caspofungin in this study were 2.9%, 5.9%, 18.8%, 47.9%, 0.0% and 16.7% in *C. tropicalis, C. glabrata* and *C. parapsilosis* respectively.

**Conclusion:**

*C. albicans* was the most prevalent species in pediatric colonized patients. New azole agents like voriconazole and posaconazole are effective against non-albicans *Candida* species. Increase in intermediate species is alarming to future emerging resistant species.

## INTRODUCTION

*Candida* species are the main cause of superficial to systemic fungal infection in humans and the major source of infection in health care centers (1). Systemic infections are common in immunocompromised pediatrics individuals including patients in hematology-oncology wards (1, 2). According to Center for Diseases Control and Prevention (CDC), *Candida* species is ranked fifth among hospital-acquired pathogens and forth among blood stream infection pathogens (3). The colonized patients are most susceptible to infection. The rates of *Candida* colonization were reported 48.8%, and 78.8% in pediatric hematologic patients (4, 5).

*C. albicans* is the main pathogenic agent of systemic infections, however, during the recent years, the rate of non *albicans Candida* species has increased in many reports (2, 5, 6). Improvement in diagnostic technical methods has led to diagnosis of other *Candida* species. And also, *C. albicans* is susceptible to most antifungal agents and during the prophylaxis cleaned from the patient’s body, but non-*albicans Candida* species like *C. glabrata* and *C. krusei* are resistant and more emerging in the infected patients.

Caggiano et al. reported “surveillance cultures are useful to monitor the *Candida* colonization in ICU patients” (7, 8). Colonization with *Candida* species is recognized as a risk factor for systemic candidiasis in immunocompromised patients (8). The susceptibility of *Candida* species varies, depending on certain species responsible for infection, geographic region, patient population and health care management in each region. Limited studies have investigated the rate of colonization and susceptibility patterns of *Candida* species isolated from colonized children. The aim of this study was to identify *Candida* species isolated from colonized hematologic pediatric patients and investigate their susceptibility patterns to seven anti-fungal agents in five university hospital centers by Clinical and Laboratory Standards Institute (CLSI).

## MATERIALS AND METHODS

Colonizing isolate was defined as *Candida* species isolated from the body site of patients without any signs and symptoms of infection.

### Sample collection.

The present study was conducted from 2014 to 2015 in order to investigate the fungal colonization from immunocompromised children admitted to five university hospitals in Iran (Shiraz, Kerman, Yasouj, Ahvaz and Sannandaj). Totally, 1950 samples were collected from mouth, nose, urine and anus. Samples were cultured on Sabouraud dextrose agar (Merck, Darmstadt, Germany) and transferred to Prof. Alborzi Clinical Microbiology Research Center for further examination. To evaluate the purity of isolates, the samples were cultured on potato dextrose agar (OXOID LTD, Basind stoke, Hampshire, England) twice at 35°C for 48h. The isolates were identified by carbohydrate assimilation reactions on API 20 C AUX system (bioMerieux, Swiss), according to the manufacturer’s instructions.

### Antifungal susceptibility testing.

The susceptibility patterns of the isolates against amphotericin B, fluconazole, ketoconazole, voriconazole, itraconazole, caspofungin and posaconazole (GmbH-Steinheim-SiGMA-Aldrichmie) were investigated using broth micro dilution assay, according to CLSI M27-A3 and S4 guidelines (9, 10). *C. parapsilosis* ATCC22019 and *C. krusei* ATCC6258 were considered as standard strains. The final concentrations of amphotericin B, itraconazole, posaconazole and voriconazole were ranged from 0.032 to 16 μg/ml and for fluconazole and caspofungin from 0.125 to 64 μg/ml and 0.016 to 8 μg/ml, respectively. In each series, one negative control without any yeast suspension and one positive control without any drugs were considered. The plates were sealed and incubated for 24 and 48h at 35°C and visual minimum inhibitory concentration (MIC) end points were determined. The recommended end-point for azole and caspofungin are the lowest drug concentration with a prominent decrease in turbidity (inhibitory concentration that gives 50% growth reduction), while for amphotericin B, MIC was the drug concentration showing a complete inhibition of growth. According to CLSI M27-A3 and S4, there is not any breakpoint for posaconazole and ketoconazole (9, 10).

### Statistical analysis.

Statistical analysis was performed using WHO NET (version 5.6). Epidemiological cutoff value (ECV), Wild-type (WT) and non-WT strain, MIC50 and MIC90 value and Geometric Mean (GM) were reported.

### Ethical considerations.

The ethics committee of Professor Alborzi Clinical Microbiology Research Center, Shiraz University of Medical Sciences reviewed and approved the study.

## RESULTS

**Fig. 1.**
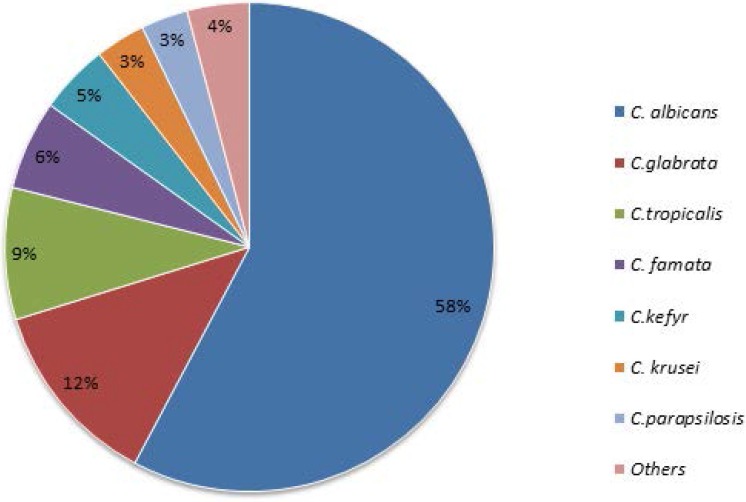
Distributions of *Candida* species isolated from pediatric patients. *Others: *C. parapsilosis, C. guilliermondii, C. lusitaniae, C. intermedia*

From 650 pediatric patients, 1950 samples were cultured and 320/650 (49.2%) were colonized with *Candida* species in different parts of their bodies and 387 *Candida* species isolated. The most prevalent backgrounds of patients were acute lymphoblastic leukemia, followed by lymphoma, and acute myelocytic leukemia (Table 1). All immunocompromised patients entered in this study had history of admission in the hospital and use of fluconazole for treatment or prophylaxis. *C. albicans* (223, 57.6%) was the most prevalent isolated species, followed by *C. glabrata* 48 (12.4%), *C. tropicalis* 34 (8.7%), *C. famata* 23 (5.9%), *C. kefyr* 18 (4.6%), *C. kuresi* 13 (3.3%), *C. parapsilosis* 12 (3.1%), *C. dubliniensis* 10 (2.5%), *C. guilliermondii* 3 (0.7%), *C. lisitaniae* 2 (0.5%) and *C. intermedia* 1 (0.25%) (Fig. 1). Distribution of *Candida* species isolated from each university hospital was shown in Table 2. According to Table 3, the sensitivity rates for *C. albicans*, the most frequently isolated species, were 99.1% to amphotericin B, 96.9% to caspofungin with 2.7% intermediate dose, 91% to voriconazole with 3.6% intermediate dose, 90.6% to fluconazole with 4.5% intermediate dose, 73.1% to itraconazole with 24.2% intermediate dose. MIC90 values for posaconazole and ketoconazole were 0.032 μg/ml and 0.125 μg/ml with GM 0.031 and 0.032, respectively. ECV in *C. albicans* for amphotericin B, caspofungin, and fluconazole were 0.25μg/ml, 0.25, and 0.5μg/ml, respectively. Few non-WT types’ *C. albicans* was isolated from the patients. According to Table 4, ECV for all *Candida* species to amphotericin B was ≤0.25 μg except *C. krusei* (4μg). The ECVs and WT rates of *C. albicans, C. parapsilosis, C. tropicalis, C. glabrata* and *C. krusei* for itraconazole were 0.5μg/ml, 96%; 0.25μg/ml, 100%; 0.5 μg/ml, 98%; 1μg/ml, 96%, 2μg/ml and 92% respectively. Other *Candida* species (*C. kefyr, C. guilliermondii, C. lisitaniae* and *C. intermedia*) were sensitive to antifungal agents and resistant rate to itraconazole in *C. famata* was 4.3%.

**Table 1.** Distributions of background illness of immunocompromised patients in five university hospitals in Iran

**Background illness**	**City**				

	**Kerman**	**Shiraz**	**Yasouj**	**Sannandaj**	**Ahvaz**
Acute lymphoblastic leukemia	28	58	26	41	20
Lymphoma	16	15	16	13	7
Acute myeloid					
leukemia	8	22	3	7	9
*Others	27	21	12	22	16
Total	79	116	57	83	52

* Others: Hodgkin’s lymphoma, Aplastic anemia, Burkit lymphoma, Yolk sac cancer, Megaloblastic anemia, Sarcoma

**Table 2.** Distributions of *Candida* species isolated from five university hospitals in the selected cities

**Species**	**No. of isolates**	**City**				

		**Kerman**	**Shiraz**	**Yasouj**	**Sannandaj**	**Ahvaz**
*C. albicans*	223 (57.6%)	41	78	26	50	28
*C. glabrata*	48 (12.4%)	8	13	12	14	1
*C. tropicalis*	34 (8.7%)	7	4	12	3	8
*C. famata*	23 (5.9%)	11	3	2	3	4
*C. kefyr*	18 (4.6%)	4	3	----	8	3
*C. krusei*	13 (3.3%)	5	6	----	2	----
*C. parapsilosis*	12 (3.1%)	1	4	1	1	5
Others	16 (4.1%)	2	5	4	2	3
Total	387	79	116	57	83	52

* Others: *C. parapsilosis, C. guilliermondii, C. lusitaniae, C. intermedia*

**Table 3.** Antifungal susceptibility patterns of *Candida* species isolated from pediatric patients by CLSI breakpoint.

**Species**	**Antifungal agents**	**Rang (μg/ml)**	**%R**	**%I**	**%S**	**MIC 50**	**MIC 90**	**Geom. Mean**
***C. albicans***	Amphotericin B	0.016–32	0.9	0	99.1	0.032	0.25	0.039
	Caspofungin	0.016–64	0.4	2.7	96.9	0.016	0.25	0.041
	Voriconazole	0.016–16	5.4	3.6	91	0.016	0.064	0.035
	Fluconazole	0.016–64	4.9	4.5	90.6	0.125	2	0.254
	Posaconazole	0.016–16	----	----	----	0.016	0.032	0.031
	Itraconazole	0.016–16	2.7	24.2	73.1	0.032	0.125	0.049
	Ketoconazole	0.016–16	----	----	----	0.016	0.125	0.032
***C. glabrata***	Amphotericin B	0.016–0.5	0	0	100	0.032	0.064	0.31
	Caspofungin	0.016–0.5	18.8	47.9	33.3	0.125	0.5	0.113
	Voriconazole	0.016–0.5	0	0	100	0.032	0.025	0.05
	Fluconazole	0.064–16	0	0	100	1	4	0.842
	Posaconazole	0.016–16	----	----	----	0.064	0.5	0.082
	Itraconazole	0.032–16	14.6	72.9	12.5	0.25	1	0.233
	Ketoconazole	0.016–16	----	----	----	0.032	0.125	0.037
***C. tropicalis***	Amphotericin B	0.016–0.5	0	0	100	0.016	0.125	0.033
	Caspofungin	0.016–4	2.9	5.9	91.2	0.032	0.25	0.046
	Voriconazole	0.016–16	8.8	5.9	85.3	0.016	0.125	0.033
	Fluconazole	0.064–64	8.8	5.9	85.3	0.125	4	0.302
	Posaconazole	0.016–16	----	----	----	0.016	0.25	0.035
	Itraconazole	0.016–16	2.9	38.2	58.8	0.064	0.5	0.078
	Ketoconazole	0.016–16	----	----	----	0.016	0.25	0.029
***C. famata***	Amphotericin B	0.016–1	0	0	100	0.032	0.25	0.037
	Caspofungin	0.016–0.25	0	0	100	0.016	0.25	0.035
	Voriconazole	0.016–1	0	0	100	0.016	0.125	0.034
	Fluconazole	0.032–8	0	0	100	0.125	4	0.268
	Posaconazole	0.016–0.5	----	----	----	0.016	0.064	0.031
	Itraconazole	0.016–1	4.3	21.7	73.9	0.064	5	0.064
	Ketoconazole	0.016–0.5	----	----	----	0.016	0.125	0.03
***C. kefyr***	Amphotericin B	0.016–1	0	0	100	0.016	0.064	0.03
	Caspofungin	0.016–2	0	0	100	0.016	0.25	0.031
	Voriconazole	0.016–0.125	0	0	100	0.016	0.032	0.021
	Fluconazole	0.064–2	0	0	100	0.125	1	0.185
	Posaconazole	0.016–0.125	----	----	----	0.016	0.032	0.021
	Itraconazole	0.016–0.25	0	22.2	77.8	0.032	0.125	0.037
	Ketoconazole	0.016–0.16	----	----	----	0.016	0.064	0.021
***C. krusei***	Amphotericin B	0.032–4	38.5	0	61.5	0.025	4	0.386
	Caspofungin	0.016–0.5	0	7.7	92.3	0.125	0.25	0.092
	Voriconazole	0.032–16	7.7	0	92.3	0.25	0.5	0.238
	Fluconazole	0.25–64	0	0	----	4	64	6.817
	Posaconazole	0.016–16	----	----	----	0.25	0.5	0.214
	Itraconazole	0.064–16	15.4	69.2	15.4	0.25	2	0.346
	Ketoconazole	0.032–16	----	----	----	0.125	4	0.33
***C. parapsilosis***	Amphotericin B	0.016–0.25	0	0	100	0.016	0.032	0.027
	Caspofungin	0.016–4	0	16.7	83.3	0.5	4	0.282
	Voriconazole	0.016–0.25	0	8.3	91.7	0.016	0.032	0.025
	Fluconazole	0.064–2	0	0	100	0.25	2	0.298
	Posaconazole	0.016–0.5	----	----	----	0.032	0.032	0.03
	Itraconazole	0.016–0.25	0	41.7	58.3	0.064	0.125	0.06
	Ketoconazole	0.016–0.032	----	----	----	0.016	0.032	0.02
***Others***	Amphotericin B	0.016–1	0	0	100	0.032	0.064	0.032
	Caspofungin	0.016–1	0	0	100	0.032	0.064	0.040
	Voriconazole	0.016–0.125	0	0	100	0.032	0.032	0.026
	Fluconazole	0.064–8	0	0	100	0.25	0.5	0.227
	Posaconazole	0.016–0.064	----	----	----	0.016	0.032	0.023
	Itraconazole	0.016–0.25	0	23.1	76.9	0.032	0.25	0.042
	Ketoconazole	0.016–0.064	----	----	----	0.016	0.032	0.022

R: Resistant, I: Intermediate, S: Susceptible, MIC: Minimum inhibitory concentration.

There is no breakpoint for Posaconazole and Ketoconazole, only MIC was reported.

**Table 4.** CLSI Clinical breakpoints and epidemiological cut off values for common *Candida* species

**Antifungal**	**Organism**	**S**	**SDD**	**I**	**R**	**ECV**	**WT**	**NWT**
**Amphotericin**	*C. albicans*	1≥	….	….	1≤	0.25	≥0.25 (98%)	>0.25 (2%)
	*C. parapsilosis*	1≥	….	….	1≤	0.25	≥ 0.25 (100%)	>0.25 (0%)
	*C. tropicalis*	1≥	….	….	1≤	0.25	≥ 0.25 (95%)	>0.25 (5%)
	*C. glabrata*	1≥	….	….	1≤	0.064	≥ 0.064 (97%)	>0.064 (3%)
	*C. krusei*	1≥	….	….	1≤	4	≥ 4 (100%)	>4 (0%)
	Others	1≥	….	….	1≤	0.064	≥0.064(92%)	>0.064(8%)
**Caspofungin**	*C. albicans*	0.25≥	….	0.5	1≤	0.25	≥ 0.25 (96%)	>0.25 (4%)
	*C. parapsilosis*	2≥	….	4	8≤	4	≥ 4(100%)	>4 (0%)
	*C. tropicalis*	0.25≥	….	0.5	1≤	0.5	≥ 0.5 (98%)	>0.5 (2%)
	*C. glabrata*	0.125≥	….	0.25	0.5≤	0.5	≥ 0.5(98%)	>0.5 (2%)
	*C. krusei*	0.25≥	….	0.5	1≤	0.5	≥ 0.5 (100)	>0.5 (0%)
	Others	2≥	….	4	8≤	0.064	≥0.064(92%)	>0.064(8%)
**Voriconazole**	*C. albicans*	0.12≥	….	0.25–0.5	1≤	0.064	≥ 0.064 (95%)	>0.064 (5%)
	*C. parapsilosis*	0.12≥	….	0.25–0.5	1≤	0.032	≥ 0.032 (91%)	>0.032 (9%)
	*C. tropicalis*	0.12≥	….	0.25–0.5	1≤	0.125	≥0.125 (92%)	>0.125 (8%)
	*C. glabrata*	ECV 0.5 μg/ml WT: MIC ≤ ECV, non-WT MIC>ECV^×^				0.25	0.25(96%)≥	>0.25 (4%)
	*C. krusei*	0.5≥	….	1	2≤	0.5	≥ 0.5 (93%)	>0.5 (7%)
	Others	….	….	….	….	0.125	≥0.125(100%)	0.125 (0%)
**Fluconazole**	*C. albicans*	2≥	….	4	8≤	8	≥ 8 (94%)	>8 (6%)
	*C. parapsilosis*	2≥	….	4	8≤	0.5	≥ 0.5 (84%)	>0.5 (16%)
	*C. tropicalis*	2≥	….	4	8≤	4	≥ 4 (92%)	>4(8%)
	*C. glabrata*	….	32≥	….	64≤	4	≥ 4 (95%)	>4 (5%)
	*C. krusei*	It is resistance to fluconazole.				64	≥64 (100%)	>64 (0%)
	Others	….	….	….	….	0.5	≥0.5(92%)	>0.5(8%)
**Itraconazole**	*C. albicans*	0.12≥	0.25–0.5	….	1≤	0.5	≥ 0.5 (96%)	>0.5 (5%)
	*C. parapsilosis*	0.12≥	0.25–0.5	….	1≤	0.25	≥ 0.25 (100%)	>0.25 (0%)
	*C. tropicalis*	0.12≥	0.25–0.5	….	1≤	0.5	≥ 0.5 (98%)	>0.5 (2%)
	*C. glabrata*	0.12≥	0.25–0.5	….	1≤	1	≥ 1 (96%)	>1 (4%)
	*C. krusei*	0.12≥	0.25–0.5	….	1≤	2	≥ 2 (92%)	>2 (8%)
	Others	0.12≥	0.25–0.5	….	1≤	0.032	≥0.032(93%)	>0.32(7%)
**Posaconazole**	*C. albicans*	There is no breakpoint, only MIC was reported.				0.25	≥ 0.25(95%)	>0.25(5%)
	*C. parapsilosis*					0.032	≥0.032(92%)	>0.032(8%)
	*C. tropicalis*					1	≥1(98%)	>1(2%)
	*C. glabrata*					1	≥1(95%)	>1(5%)
	*C. krusei*					0.5	≥0.5(93%)	>0.5(7%)
	Others					0.064	≥0.064(100%)	>0.064(0%)
**Ketoconazole**	*C. albicans*	There is no breakpoint, only MIC was reported.				0.25	≥0.25(95%)	>0.25(5%)
	*C. parapsilosis*					0.032	≥0.032(100%)	>0.032(0%)
	*C. tropicalis*					0.5	≥0.5(97%)	>0.5(3%)
	*C. glabrata*					0.125	≥0.125(96%)	>0.125(4%)
	*C. krusei*					16	≥16(100%)	>16(0%)
	Others					0.032	≥0.32(93%)	>0.32(7%)

S: Susceptible, SSD: Susceptible dose dependent, I: Intermediate, R: Resistant, ECV: Epidemiological cut off value; WT: Wild type, NWT: Non-wild type

## DISCUSSION

For the management of systemic candidiasis in immunocompromised patients, early diagnosis and empirical antifungal therapies are in focus. Leon et al. reported multifocal colonization (OR=3.04, 95% CI, 1.45–6.39) was predictive of proven *Candida* infection and would benefit from early antifungal therapy (11). As the colonized *Candida* may transfer to pathogen due to change in patients’ immune system, knowledge about identification and antifungal susceptibility patterns of colonized organism can be helpful for best therapy and less resistance. *C. albicans* was the most prevalent species in all cities. *C. glabrata* was the second isolate from Shiraz, Yasouj and Sannandaj but *C. famata* and *C. tropicalis* were the second isolates from Kerman and Ahvaz. The prevalence rate of *C. albicans* in different studies were reported 48.6% (172/354) (12), 51.2% (117/229) (13) and 79.1% (53/67) (14). Emergence of non-*albicans* species in recent decades has been rising. The most prevalent non-*albicans Candida* isolates in the present study were *C. glabrata, C. tropicalis, C. famata, C. kefyr* and *C. krusei* (Table 2). In our study, 43.1% of all *Candida* isolates was non *albicans* species, while the rates in other studies were reported 48.8 % (13), 45% (15) and 21.8% (16). In the study conducted by Wisplinghoff et al. *C. parapsilosis* (17.4%), *C. glabrata* (16.7%) and *C. tropicalis* (10.2%) were responsible for bloodstream infections (18). The sensitivity rates of 178 *C. albicans* isolated from immunocompromised patients were reported 93%, 95.4%, 93% and 97.7% for amphotericin B, fluconazole, itraconazole, and voriconazole, respectively (17). In another study by Moran et al. “Children with non-albicans bloodstream infections were approximately twice as likely to die as children with *C. albicans* bloodstream infections (35.2% versus 18.2%; P= 0.03)”(19). The mortality rates of non *albicans Candida* bloodstream infection in children were reported 29.7%, 41.7% and 57.1% for *C. parapsilosis, C. tropicalis* and *C. glabrata*, respectively (19). Distributions of *Candida* species are different according to region and patient’s populations. Therefore, identification of *Candida* species isolated from pediatric patients is valuable in each region.

Amphotericin B is a common antifungal agent recommended for fungal infection therapy but its use has some limitations due to the risk of toxicity. In the present study, most of *Candida* species isolates were susceptible to amphotericin B except *C. albicans* and *C. krusei* with resistance rates of 0.9% and 38.5%, respectively.

In the *Candida* species isolated from immunocompromised patients the resistance rates to amphotericin B were reported 7% (12/172) in *C. albicans*, 10% (6/62) in *C. krusei*, 15% (6/40) in *C. glabrata*, 22.3% (4/18) in *C. parapsilosis* and 33.3% (2/6) in *C. tropicalis* (12). While these rates in colonized pediatric patients were reported 3.4% (4/117), 27.7% (5/18) and 7.1% (1/14) in *C. albicans, C. krusei* and *C. glabrata*, respectively (13).

Fluconazole is a triazole agent that is the most prescribed antifungal agents for the treatment of *Candida* infections. Other azoles antifungal agents include voriconazole, posaconazole and itraconazole. The resistance rate to fluconazole in this study in *C. albicans* was 4.9% with MIC90 value 2 μg/ml and ECV 8 μg/ml, followed by *C. tropicalis* 8.8%, MIC90 value 4 μg/ml and ECV 0.5 μg/ml. The resistance rates in *C. albicans* to fluconazole were reported 12% (14/117) in colonizing isolates in neutropenic patients, 9.3% (16/354); and 81% (43/53) in infecting isolates (12–14). In Wisplinghoff et al. report 100% of *C. glabrata*, 4.9% of *C. tropicalis*, 2.9% of *C. parapsilosis* and 0.8% of *C. albicans* were not susceptible to fluconazole (18). The resistance rate of *C. glabrata* to fluconazole was reported 36% with 64% susceptible dose dependent (20). The acquired resistance to fluconazole (29.4%; P<0.05) is reported in *C. glabrata* isolates from colonized oral cavity in patients exposed to azoles (21). The increase resistance rate of *Candida* species to fluconazole maybe due to the frequent use of its medication. Voriconazole is an active azole antifungal agent against *Candida* species. In the present study, its susceptibility rate in *C. glabrata* and *C. krusei*, as the resistant *Candida* species, were 100%, (MIC50: 0.032 μg and MIC90: 0.016μg), and 92.3% (MIC50: 0.25 and MIC90: 0.5), respectively. The non-susceptible rates of *Candida* species to voriconazole were reported 9.8% of *C. tropicalis*, 7.6% of *C. parapsilosis*, 5.0% of *C. krusei* and 0.6% of *C. albicans* (18). In study done by pfaler et al., only *C. krusei* was resistant to voriconazole and other *Candida* species were susceptible to it (22). There is no breakpoint for posaconazole and ketoconazole, according to CISI M27 S4(10). Posaconazole is the newest triazole antifungal and very expensive in our region. All *Candida* species had MIC value between 0.032 and 0.5 μg/ml. *Candida glabrata* MIC90 values for posaconazole and ketoconazole were 0.5 μg/ml and 0.125 μg/ml with GM 0.082 μg/ml and 0.037 μg/ml, respectively. MIC90 value and GM for *C. tropicalis* to posaconazole and ketoconazole were 0.25 μg/ml and 0.25 μg/ml; and 0.035 μg/ml and 0.029 μg/ml, respectively. The MIC values for posaconazole were reported 0.016 μg/ml, 0.25 μg/ml, 0.125 μg/ml and 0.5 μg/ml in *C. albicans, C. tropicalis, C. parapsilosis* and *C. glabrata*, respectively (23). Posaconazole and voriconazole are used limitedly and are effective on *Candida* species isolates from the patients.

The sensitivity rates of *C. glabrata* and *C. krusei* to itraconazole were 12.5% (with 72.9% intermediate dose) and 15.4% (with intermediate dose 69.2%), respectively. The resistance rates to itraconazole in colonized species were reported 28% (36/117), 30% (6/18) and 50% (7/14) in *C. albicans, C. krusei* and *C. glabrata*, respectively (13). These rates were reported 86% (80/93), 59.5% (25/42) and 7.7% (2/26) in *C. albicans, C. glabrata* and *C. parapsilosis*, respectively (24). The increase in intermediate rates is alarming for future resistant strains. Ketoconazole is mostly used as a topical due to its side effects for humans. In the present study, the MIC90 values for all *Candida* species to ketoconazole were ≤0.25μg/ml except *C. krusei* which was 4 μg/ml. The resistance rates for this drug in *C. glabrata* and *C. albicans* were reported 33.3% (14/42) and 17.2% (16/93) respectively (24).

Of the echinocandin antifungal agents, caspofungin is more prescribed in our region. The resistant and intermediate rates of *Candida* species to caspofungin in this study were 0.4% and 2.7%; 2.9% and 5.9%; 18.8% and 47.9%; and 0.0% and 16.7% in *C. albicans, C. tropicalis, C. glabrata* and *C. parapsilosis*, respectively. Other *Candida* species were susceptible to caspofungin. *C. krusei* has intrinsic resistance to fluconazole and has been shown the highest sensitivity to caspofungin and voriconazole (S=93.3%). In Korean patient, none of the *Candida* species was resistant to caspofungin (25).

## CONCLUSiON

Colonizing *Candida* species may be present as reservoir for future systemic candidiasis. In the present study, 49.2% of pediatric patients wit hematologic disorders were colonized with *Candida* species. *C. albicans* was the most prevalent species in pediatric colonized patients. New azole agents like voriconazole and posaconazole are effective to non-*albicans Candida* species. Increase in intermediate species is alarming to future emerging resistant species. The information about distribution and susceptibility patterns of species can be useful to appropriate treatment in hematopoietic pediatric patients at the duration of infection when sampling is impossible.
